# Fine-grained structural classification of biosynthetic gene cluster-encoded products

**DOI:** 10.1093/bioinformatics/btag559

**Published:** 2026-08-17

**Authors:** Vladimir Porokhin, Emily Mevers, Justin J J van der Hooft, Soha Hassoun

**Affiliations:** Department of Computer Science, Tufts University, Medford, MA 02155, United States; Department of Chemistry, Virginia Tech, Blacksburg, VA 24061, United States; Bioinformatics Group, Wageningen University & Research, Wageningen 6708PB, The Netherlands; Department of Biochemistry, University of Johannesburg, Johannesburg 2006, South Africa; Department of Computer Science, Tufts University, Medford, MA 02155, United States; Department of Chemical and Biological Engineering, Tufts University, Medford, MA 02155, United States

## Abstract

**Motivation:**

Biosynthetic gene clusters (BGCs) are responsible the biosynthesis of many natural products, including a multitude of effective therapeutics and their precursors. Advances in genomic data collection as well as computational techniques have made it possible to identify BGCs at scale. However, accurately determining the types of BGC-encoded products from genomic content remains elusive.

**Results:**

Here, we introduce BGC annotation tool (*BGCat*), a machine learning method for fine-grained structural classification of BGC-encoded products, leveraging the NPClassifier natural product nomenclature. Our method leverages a pre-trained protein language model for creating meaningful gene representations and a deep neural network for class label prediction. We show the method outperforms state-of-the-art approaches in coarse-grained product classification and is effective for detailed classification. We implement a clustering-based augmentation strategy for BGC-product relationships, addressing a crucial gap in the available datasets. We then introduce the concept of product class profiles of gene cluster families (GCFs), associating each GCF with a probabilistic distribution of product types and offering a new perspective on GCF functions. Lastly, we use *BGCat* to provide new product class labels for over 100k BGCs in antiSMASH DB that presently have minimal information about their products.

**Availability and implementation:**

The source code and trained model weights are freely available at https://github.com/HassounLab/BGCat.

## Graphical Abstract

**Graphical Abstract:**
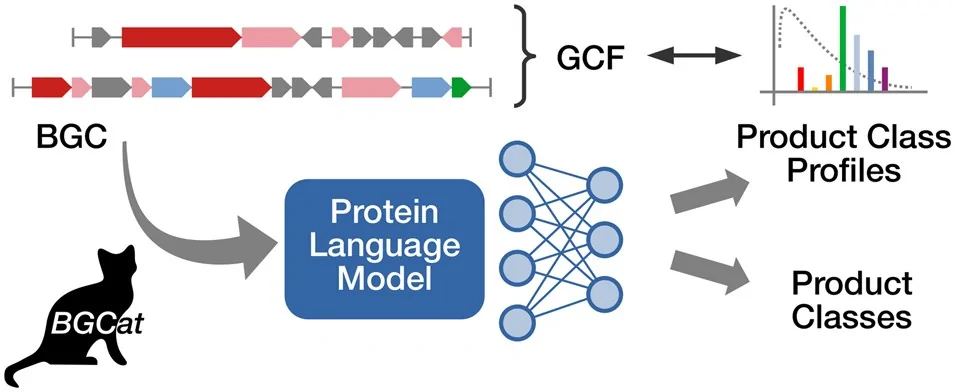

## 1 Introduction

Natural products have been a staple of medicine for much of human history. Natural products represent a vast reservoir of potential drug candidates, with a high proportion of biologically active species (Demain 2014, Newman and Cragg 2020). A large percentage of drugs in current use owe their existence to natural products, including antibiotic lariocidin (Jangra *et al.* 2025), anti-tumor agent anthramycin (Leimgruber *et al.* 1965), antiparasitic drug Ivermectin (Shen 2015), and ∼70% of anti-infective drugs (Chen *et al.* 2019). By fall of 2019, over a third (38%) of FDA-approved new molecular entities were either natural products or their derivatives, and in select areas (e.g. cancer drugs) that percentage can be as high as 65% (Newman and Cragg 2020). As such, there is an ongoing interest in natural products and their sources.

In microbes, the specialized genes that are used to synthesize natural products are clustered into biosynthetic gene clusters (BGCs). These genes encode enzymes that synthesize and tailor the structure of the product, as well as regulatory, and transport proteins involved in its synthesis (Kjærbølling *et al.* 2019). The recent explosion of microbial genomic data has led to the development of computational techniques for identifying BGCs. Early methods relied on sequence alignment algorithms and manually curated rules. However, with the rapidly growing quantity of identified BGCs and their diversity, algorithmic and rule-based approaches have given the way to machine learning techniques, e.g. DeepBGC (Hannigan *et al.* 2019), BiGCARP (Rios-Martinez *et al.* 2023), and BGCCGB (Du *et al.* 2024), promising a more flexible and comprehensive framework for understanding BGCs. AntiSMASH (ANTIbiotics and Secondary Metabolite Analysis SHell), currently the most widely used tool for mining microbial genomes for BGCs (Blin *et al.* 2023a), leverages profile Hidden Markov Models in conjunction with a bank of rules for detecting and characterizing BGCs, with the latest version supporting up to 101 cluster types (Blin *et al.* 2025).

However, despite advances in BGC detection, determining the identities of the natural products encoded by BGCs remains challenging. For example, although antiSMASH infers the approximate scaffold for some metabolites based on polyketide synthase (PKS) and non-ribosomal peptide synthase (NRPS) domains and includes some analysis features for tailoring enzymes (Blin *et al.* 2025), it currently does not predict the full product structure with tailoring modifications. Several methods were proposed for product structure prediction, including RiPPMiner (Agrawal *et al*. 2017), TransATor (Helfrich *et al.* 2019), and PRISM (Skinnider *et al.* 2020); however, they are either limited to specific types of BGCs or are unable to recognize novel enzymatic activities, restricting their general applicability. Recently, CHAMOIS was introduced as a generalized model for characterizing chemical properties of BGC products based on Pfam domains (Larralde and Zeller 2025); however, its ClassyFire-based (Dührkop *et al.* 2021) classification system was primarily designed for the wider bio-organic chemistry community and as such is less than ideal for natural products (Kim *et al.* 2021). Meanwhile, the traditional approach to BGC product classification, as seen in DeepBGC (Hannigan *et al.* 2019), BiGCARP (Rios-Martinez *et al.* 2023), and BGCCGB (Du *et al.* 2024), uses a coarse-grained set of classes provided in MIBiG (Minimum Information about a BGC) (Zdouc *et al.* 2025). This nomenclature currently categorizes products into seven classes: Alkaloids, Polyketides, NRPs (non-ribosomal peptides), RiPPs (ribosomally synthesized and post-translationally modified peptides), Saccharides, Terpenes, and Others. Because these approaches rely on broad, coarse product categories, they offer limited insight into the structural diversity of the encoded metabolites. This gap highlights the need for fine-grained structural classification methods capable of distinguishing natural products at a resolution meaningful for biosynthetic and chemical analysis.

The lack of high-quality data associating BGCs and natural products has been a major obstacle for gleaning insight into product structures. For example, the largest repository of such information, MIBiG, provides a selection of 3013 BGCs (Zdouc *et al.* 2025). Consequently, a common strategy has been to group BGCs into gene cluster families (GCFs), which can link BGCs to better-annotated MIBiG entries that include structural information as a starting point for BGC characterization (Kautsar *et al.* 2021a, 2021b). Implementations of this approach typically rely on network analysis (Cimermancic *et al.* 2014), sequence similarity (Doroghazi *et al.* 2014, Leao *et al.* 2017), or a combination thereof (Navarro-Muñoz *et al.* 2020, Kautsar *et al*. 2021b), with statistical correlation techniques sometimes introduced to leverage mass spectrometry data (Doroghazi *et al.* 2014, Goering *et al.* 2016). Grouping clusters in such fashion provides important context for understanding BGCs and has been shown to create meaningful families that are comparable to chemical classes (Kautsar *et al*. 2021b). However, the focus thus far has been on establishing connections to known clusters as opposed to learning more general patterns of BGC product expression.

We introduce in this paper *BGCat* (BGC annotation tool), a technique for fine-grained structural classification of BGC-encoded natural products following the NPClassifier nomenclature designed for natural products (Kim *et al.* 2021). Our technique takes a set of genes comprising a BGC and outputs a set of predicted likelihoods for each type of natural product; the higher the likelihood, the stronger the association between that BGC and product type. A given BGC may be associated with one or more types. *BGCat* leverages ESM Cambrian, a protein language model trained on a wide range of protein sequences (ESM Team 2024) (https://evolutionaryscale.ai/blog/esm-cambrian), as the engine for condensing relevant biosynthetic genes into meaningful embeddings. We show that our approach surpasses the state-of-the-art techniques for traditional coarse-grained BGC product classification. Next, we demonstrate that our technique is effective for detailed product classification. We then introduce a clustering-based augmentation method that provides additional data for model training and enhances its performance. Next, we investigate product class profiles (PCPs) of GCFs to help identify the most meaningful families. Finally, we leverage *BGCat* to introduce detailed product labels for thousands of BGCs in the antiSMASH database (antiSMASH DB) (Blin *et al.* 2023b), including the many clusters with unknown products. We make our model available at https://github.com/HassounLab/BGCat.

## 2 Methods

### 2.1 Dataset construction

Our dataset is derived from MIBiG, the largest manually curated source of experimentally validated BGCs along with their products. The database is supported by an online community of 250+ members and currently includes 3013 clusters (Zdouc *et al.* 2025), 96% of which are associated with bacteria and fungi. To use the most reliable BGC annotations, we exclude 377 of those clusters that are marked as retired, resulting in a set of 2636 MIBiG BGCs. Next, we leverage NPClassifier (Kim *et al.* 2021) to obtain detailed product type labels for these BGCs. The nomenclature used by NPClassifier is designed to be informative for natural products and features a hierarchy with three distinct levels: 7 pathways, 70 superclasses, and 672 classes; however, not all of them are covered by the BGC products. The product structures are processed as SMILES strings, which we canonicalize and de-duplicate using RDKit (RDKit Contributors 2022) to ensure each unique product is only classified once. When a BGC is known to produce multiple products, their labels are combined in a multi-label fashion. This process results in a dataset associating BGCs and their corresponding product classifications. In the set of MIBiG BGCs, 521 clusters lacked machine-readable product structures (e.g. a valid SMILES string was not specified) and an additional 69 BGCs produced no NPClassifier predictions; both of these BGC types were excluded from further consideration. This process yielded 2046 BGCs, featuring 3479 product structures, together resulting in 3839 unique BGC-product pairs that formed our core MIBiG dataset. The most common compound categories at the pathway level were Polyketides and Amino acids & Peptides, with 77% and 65% of the BGCs featuring at least one product of the respective type. On average, each product was associated with 1.15 unique pathway labels.

### 2.2 Model architecture and training

**Figure 1:**
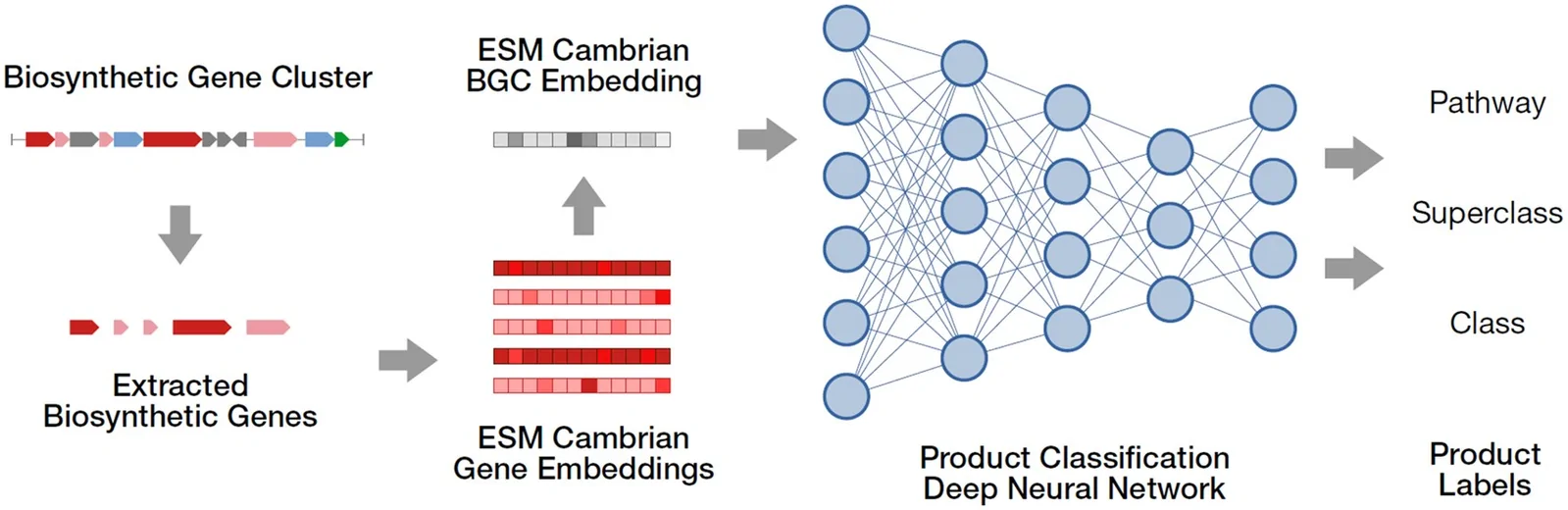
An overview of the *BGCat* model. A BGC identified by antiSMASH or a similar tool is accepted as the input. Next biosynthetic genes are extracted and embedded using ESM Cambrian. Regulatory, transport-related, and other genes are ignored. The gene-level embeddings are then aggregated to yield an embedding representative of the BGC. A deep neural network is then used to predict pathway, superclass, and class labels of the potential products.

We structured our model as a feed-forward deep neural network. The network operates on BGC gene embeddings and predicts product classification in a multi-label fashion. We implemented three separate networks for each of the pathway, superclass, and class label types, as well as a combined network that predicts all three types simultaneously. The BGC embeddings are constructed by applying the ESM Cambrian 600M (ESM Team 2024) model to all biosynthetic (core and additional) genes; the remaining gene types (regulatory, transport-related, and other) are excluded due to their incidental effect on the product structure and to minimize the likelihood of considering unrelated genes that may be included in a BGC due to lenient distance thresholds used by antiSMASH for cluster detection (Blin *et al.* 2019). The exclusive focus on biosynthetic genes aims to capture the essential product features; however, it may also miss some of them due to antiSMASH not always being able to predict all such genes. The genes are encoded as sequence tokens, each representing one of the 20 standard amino acids, using the default ESM Cambrian tokenizer. Mean pooling is then used to combine gene-level embeddings into whole-BGC embeddings. The input dimension of the network is 1152 to match the size of the BGC embeddings, the hidden layers progressively reduce in their dimensionality, and the final layer predicts the target labels. The model consists of three hidden layers implemented using PyTorch and is trained using the Adam optimizer (Kingma and Ba 2017) with binary cross-entropy as the loss function. The architecture of the model is illustrated in Fig. 1.

### 2.3 Dataset augmentation

The core MIBiG dataset was augmented with BGCs sourced from BGC Atlas (Bağcı *et al.* 2025), an online database containing nearly 1.8 million clusters, derived from 35k bacterial metagenomes found in MGnify (Richardson *et al.* 2023). These BGCs were identified by antiSMASH and as such include both complete and incomplete clusters. Incomplete BGCs pose a problem in this instance as they may be insufficient to support product classification; we therefore focused on the subset of 204 661 complete clusters. In addition, the BGC Atlas clusters provide limited details about their product molecules, so we sought to leverage the product information available in similar BGCs in MIBiG. Following the method described in BGC Atlas, we grouped the complete BGC Atlas clusters into GCFs using BiG-SLiCE 2.0.0 (Kautsar *et al*. 2021b) with a distance threshold of 0.4, resulting in a set of 18 596 GCFs. Next, we queried our core MIBiG BGCs against those GCFs, finding at least one similar MIBiG BGC for 743 GCFs from the set. In the event BiG-SLiCE identified multiple candidate GCFs for a given BGC, only the highest-ranking assignment was used. We assume that all BGCs within a given GCF will yield substantially similar products; therefore, product classification labels from the MIBiG BGCs were copied to all BGC Atlas-derived BGCs that are part of the same GCF. This process resulted in a set of 9055 BGCs sourced from BGC Atlas with their corresponding product labels. These BGCs, combined with our core MIBiG set, resulted in 11 101 BGC-label pairs which we refer to as our augmented set.

## 3 Results

### 3.1 Method outperforms existing techniques in traditional BGC product classification

Currently there is no comparable approach that seeks to classify BGC products based on a detailed nomenclature appropriate for natural products. Most of existing work in this space treats BGC product classification as an incidental problem to BGC detection, classifying products into the seven coarse-grained classes provided in MIBiG (Zdouc *et al.* 2025). Although this nomenclature provides somewhat limited information about the product, it offers a benchmark where we can compare against current baseline techniques. We considered four methods as our baselines: the built-in classification functionality of antiSMASH (Blin *et al.* 2025), DeepBGC (Hannigan *et al.* 2019), BiGCARP (Rios-Martinez *et al.* 2023), and BGCCGB (Du *et al.* 2024). We evaluated the model on two versions of the MIBiG database: version 1.4 to allow for direct comparison with previous work, and version 4.0 to use the most up-to-date information. In both cases, the evaluation was performed using 5-fold cross-validation, which aims to offer a more reliable performance estimate by averaging results across multiple training-test splits. Each split is assembled from folds, where one is held out as a test set and the remaining folds are used for training; the folds were randomly chosen at the start of the procedure and all five possible training-test splits were considered. The MIBiG data consists of human-annotated BGCs observed in nature, ensuring that the model’s predictions are grounded in real world data.

As shown in Table 1, our approach provides substantial performance gains over the baselines in traditional BGC product classification. In a head-to-head comparison using the MIBiG v. 1.4 database, our method improved upon AUROC and precision relative to prior approaches, although the recall was lower than that of BGCCGB. However, this gap in recall disappeared when the model was evaluated on MIBiG v. 4.0. These trends help illustrate two key areas where our method has improved over existing techniques. First, the use of ESM Cambrian embeddings of gene sequences alongside a deep neural network resulted in substantial gains over all but one baseline methods. Second, the introduction of additional BGC information available in a more recent release in MIBiG allowed our model to better learn the product classification task and surpass all baselines.

**Table 1:** Average AUROC, recall, and precision for traditional BGC product classification; table adapted from BGCCGB (Du *et al.* 2024).

Metric	antiSMASH	DeepBGC	BiGCARP	BGCCGB	Our method with	
					MIBiG 1.4	MIBiG 4.0
AUROC	0.784	0.792	0.855	0.862	0.936	**0.937**
Recall	0.718	0.691	0.539	0.740	0.693	**0.768**
Precision	0.658	0.741	0.593	0.732	0.811	**0.825**

The highest values are shown in bold.

There are additional models capable of coarse-grained classification, including the recently debuted BGC-MAC (Lu *et al.* 2026) and BGC-Prophet (Lai *et al.* 2025). However, these methods were benchmarked under different conditions, making comparisons with *BGCat* difficult. For example, BGC-MAC reports its performance on a per-class basis, using a distinct held-out fold as its test set. While ballpark figures can be estimated by the means of weighted averaging (suggesting AUROC of 0.953, recall of 0.887, and precision of 0.825), they are not directly comparable to our reported results. Meanwhile, BGC-Prophet reports its performance based on more targeted genomic datasets that do not correspond to MIBiG.

### 3.2 *BGCat* is effective for BGC natural product class prediction

Next, we apply our method for detailed natural product class prediction. In this problem, we consider the three NPClassifier classification levels with increasing level of specificity: pathway, superclass, and class (Kim *et al.* 2021). The pathway level consists of seven classes: Alkaloids, Amino acids and Peptides, Carbohydrates, Fatty acids, Polyketides, Shikimates and Phenylpropanoids, and Terpenoids. As such, pathway-level classification is similar to MIBiG class prediction seen in traditional BGC product classification, although the mapping between the two is not one-to-one. Meanwhile, the set of possible superclasses and classes is substantially larger, with an almost order of magnitude increase at each level. We consider two types of models: one that predicts a specific level of classification, and another that predicts all levels simultaneously.

To provide a comprehensive evaluation of the method, we consider four metrics: average precision (AP), area under the receiver operating characteristic (AUROC), recall, and precision. The latter two provide an approximate measure of performance assuming a fixed likelihood threshold of 0.5, which is a customary default choice (Freeman and Moisen 2008) for binary classifiers (e.g. whether or not a BGC makes a product of a particular class). However, such a cut-off is not necessarily optimal and its selection is subject to trade-offs (Freeman and Moisen 2008, Parker 2011). Therefore, we also consider AUROC, a traditional classifier performance metric, as well as average precision, a measure of the quality of relative ranking. A key characteristic of our BGC dataset is class imbalance, with some types of BGC products being vastly overrepresented compared to others. We therefore use two averaging schemes for our metrics. Micro averaging treats each prediction in a standalone fashion, representing the performance of a typical prediction. Macro averaging computes the metric for each class separately and reports the average across the classes. With all classes being treated equally irrespective of any imbalance, macro averaging represents the performance of the method for a randomly selected class. When applied to average precision, this macro-averaged value corresponds to the mean average precision (mAP) commonly used in multi-label classification.

The results calculated with 5-fold cross-validation on the MIBiG 4.0 set can be found in Table 2. We compare our method with a baseline Random Forest (RF) classifier based on the configuration featured in DeepBGC (Hannigan *et al.* 2019) and MultiOutputClassifier from the scikit-learn python package, achieving results shown in Table 2 (a) for the baseline method and Table 2 (b) for *BGCat*. Our approach significantly improves upon the performance of the RF classifier, with the exception of a slight performance penalty in precision. The method achieves high AUROC values across the board; however, other measures are more varied. In general, performance reduces with increasingly more specific classification levels. The prediction of all levels simultaneously presents intermediate performance.

**Table 2:** NP label prediction performance, for all labels being predicted at the same time and pathway, superclass, and class labels being predicted separately.^a^

Label type	Micro averaging				Macro averaging			
	AP	AUROC	Recall	Precision	AP	AUROC	Recall	Precision
(a) Random Forest NP prediction with MIBiG 4.0								
All labels	0.286	0.662	0.324	0.847	0.070	0.531	0.063	0.855
Pathway	0.554	0.771	0.562	0.850	0.403	0.673	0.374	0.881
Superclass	0.259	0.639	0.279	0.872	0.086	0.538	0.076	0.882
Class	0.107	0.562	0.125	0.793	0.049	0.522	0.045	0.822
(b) BGCat NP prediction with MIBiG 4.0								
All labels	0.606	0.930	0.397	0.836	0.302	0.810	0.040	0.815
Pathway	0.854	0.939	0.771	0.829	0.741	0.910	0.642	0.784
Superclass	0.673	0.929	0.467	0.866	0.511	0.852	0.182	0.881
Class	0.362	0.876	0.160	0.781	0.300	0.793	0.016	0.807
(c) Temporal split on MIBiG								
All labels	0.655	0.923	0.444	0.874	0.324	0.780	0.071	0.759
Pathway	0.898	0.956	0.811	0.852	0.777	0.920	0.654	0.882
Superclass	0.674	0.905	0.479	0.849	0.460	0.757	0.195	0.834
Class	0.423	0.863	0.256	0.795	0.310	0.778	0.031	0.810

a (a, b) Results for a random 5-fold cross-validation split for the Random Forest baseline and *BGCat*, respectively. (c) Results for a temporal split on MIBiG, with updates between versions 3.1 and 4.0 used as the test set.

Recall values are considerably lower with the more detailed superclass- and class-level predictions, suggesting that the model has more limited information about those labels and tends to assume the more common case that a given BGC is not associated with a particular detailed label. This is likely a consequence of class imbalance, as implied by the further diminished macro averaging results, where the underrepresented classes have greater impact on the metric. However, precision remains high, indicating that the model is relatively accurate in the positive predictions it makes. It should be noted that because the dataset is stratified by BGCs, there is a high possibility for data leakage in this “overall” performance evaluation. For example, aflatoxin biosynthesis is represented by multiple clusters in MIBiG, which may allow that biosynthetic function to appear in both the training and test sets. To provide a more rigorous evaluation we therefore consider additional data splits.

To showcase the performance of the method as additional BGC information becomes available, we introduce a temporal data split. In this instance, we retained BGCs in a previous version of MIBiG (v. 3.1) for training the model, and reserved the BGCs added in the next version (v. 4.0) for testing the performance. The results can be found in Table 2 (c). In this case, the performance of the method is broadly similar to that with random cross-validated split; however, some metrics are varied likely due to the smaller test set size: the test size in the cross-validated split was 768, while in the temporal split it was 183. Nevertheless, this indicates the method remains effective on newly added BGCs.

### 3.3 Dataset augmentation enhances BGC NP class predictions

The core MIBiG dataset is restrictive for machine learning methods that require large amounts of data. As such, we sought to increase the number of training examples by introducing a data augmentation scheme that has expanded the dataset size 5-fold. The effect of that change on prediction performance can be seen in Table 3 (a). The evaluation was performed on the same temporal split as in Table 2 (b), and the BGCs reserved for testing were excluded from the augmentation scheme. We note a consistent increase in all metrics except for precision. This indicates the model was able to recall more of the classes with enhanced quality of ranking, at the expense of slightly lower accuracy of its predictions.

**Table 3:** NP label prediction performance with dataset augmentation.^a^

Label type	Micro averaging				Macro averaging			
	AP	AUROC	Recall	Precision	AP	AUROC	Recall	Precision
(a) Temporal split								
All labels	0.669	0.937	0.554	0.784	0.426	0.854	0.235	0.750
Pathway	0.889	0.962	0.837	0.809	0.760	0.936	0.689	0.783
Superclass	0.637	0.924	0.521	0.731	0.436	0.856	0.310	0.601
Class	0.495	0.896	0.413	0.735	0.361	0.835	0.196	0.796
(b) Augmentation split								
All labels	0.442	0.933	0.508	0.404	0.135	0.819	0.078	0.238
Pathway	0.596	0.871	0.742	0.481	0.453	0.824	0.599	0.408
Superclass	0.403	0.915	0.459	0.337	0.177	0.832	0.144	0.248
Class	0.201	0.894	0.270	0.312	0.124	0.810	0.054	0.300
(c) Realistic split								
All labels	0.315	0.835	0.271	0.531	0.112	0.646	0.049	0.455
Pathway	0.527	0.790	0.505	0.530	0.403	0.734	0.367	0.443
Superclass	0.304	0.807	0.242	0.562	0.129	0.691	0.067	0.386
Class	0.151	0.772	0.127	0.448	0.094	0.628	0.032	0.452
Alkaloids (2096)	0.034	0.700	0.020	0.175	0.077	0.650	0.013	0.217
Amino Acids & Peptides (4465)	0.275	0.856	0.221	0.493	0.183	0.637	0.090	0.567
Carbohydrates (1018)	0.068	0.713	0.028	0.375	0.079	0.717	0.025	0.188
Fatty Acids (393)	0.029	0.683	0.027	0.148	0.021	0.553	0.010	0.095
Polyketides (1459)	0.148	0.775	0.141	0.410	0.153	0.745	0.053	0.537
Shikimates & Phenylpropanoids (2680)	0.180	0.675	0.147	0.667	0.057	0.494	0.039	0.386
Terpenoids (293)	0.029	0.561	0.008	1.000	0.047	0.560	0.003	1.000

a (a) Results for a temporal split on MIBiG, with updates between versions 3.1 and 4.0 used as the test set. (b) Results for the augmentation split, where the augmented examples were used for training and non-augmented ones for testing. (c) Results for the realistic data split, with larger GCFs used for training and smaller unseen GCFs for testing. The number of BGCs in the training set for specific pathways is shown in parentheses.

Next, we validated whether the BGCs introduced by augmentation were informative by introducing an augmentation split, where the clusters sourced from BGC Atlas were used for training the model and the remaining ground-truth MIBiG BGCs were reserved for testing. The results can be found in Table 3 (b). With lack of access to high-quality MIBiG BGCs, the performance of the method has significantly decreased; however, it remained effective to a degree, suggesting that the added BGCs provide valuable information for the product classification task.

### 3.4 Model generalizes to unseen GCFs

With the landscape of known and well-annotated BGCs being limited, it is highly desirable for the model to be applicable to novel BGCs with no previously described function. To estimate the performance of the method under those circumstances, we introduce a “realistically novel” data split similar to one described in Profile-QSAR (Martin *et al.* 2017). This split seeks to maximize the difference between training and test sets by separating BGCs according to their GCF membership. The training set is assembled from the BGCs comprising the largest GCFs, while the smallest GCFs are used to build the test set. This approach reflects a degree of realism because the largest GCFs are likely to contain more common BGCs with a greater likelihood of appearing in our dataset. Conversely, the likely less common BGCs in the smallest GCF are reflective of novel BGCs that we might not encounter in the course of training our model. In addition, this realistic split reduces the possibility of data leakage due the the same biosynthetic function occasionally appearing as multiple BGCs in MIBiG: such BGCs are likely to be grouped within the same GCF that would be wholly allocated to either the training or the test set. As such, this evaluation may provide a more practical measure of performance and insight into the model’s generalizability.

The results of this experiment can be found in Table 3 (c). Compared to the temporal split in Table 3 (a), the performance is considerably lower; however, the model retains a degree of effectiveness on the unseen GCFs, demonstrating its generalizability.

To gain a better understanding of the strengths and limitations of the method, we explore the relative difficulty of predicting detailed product classes under each pathway. Compared to the overall baseline, the classes grouped under Amino Acids and Peptides present substantially higher performance, followed by Shikimates and Phenylpropanoids. This is most likely a consequence of having a larger training set for these pathways, 4465 and 2680 BGCs, respectively. The other pathways are represented by 2096 BGCs or fewer in the training set, making it more difficult for the model to learn the necessary predictive patterns. Nevertheless, even with as few as 293 BGCs in the Terpenoid training subset, the model is able to predict some product classes accurately.

Because the dataset exhibits significant class imbalance, we also consider training the model with a loss function that treats all classes equally. In particular, we use binary cross-entropy loss where the weights of positive examples for a given class *c* were adjusted by a factor $N_{c}^{-}/N_{c}^{+}$, where $N_{c}^{-}$ and $N_{c}^{+}$ represent the numbers of negative and positive examples for class *c*, respectively. However, this change appears to have minimal effect on method performance. For more details, see Table 1, available as supplementary data at *Bioinformatics* online.

### 3.5 BGC product classification offers a new perspective on GCFs

Detailed classification of BGC natural products enables more comprehensive understanding of GCFs. Existing methods for constructing GCFs primarily leverage genomic sequence information for grouping similar BGCs, and although they do not directly consider the natural products, the resulting families are often correlated with specific product structures (Cimermancic *et al.* 2014, Doroghazi *et al.* 2014). However, it remains uncertain whether this pattern holds universally for all types of products and BGCs. As such, there is value in considering the natural products more explicitly.

To offer greater insight into GCFs, we propose the concept of PCPs, whereby each GCF is associated with a probability distribution of natural product types. We construct these distributions accordingly, by applying our model to these BGCs and aggregating the predicted types for each GCF. Thus, a given GCF may be associated with specific classes of molecules to varying degrees. Of particular interest are the GCFs with a distinct PCP, whose product type distribution is meaningfully different from the overall distribution of the dataset (the background), as such GCFs are likely to be informative: e.g. they may be strongly associated with particular types or have unique characteristics that are not captured by the clustering metric. We identify these GCFs using Pearson’s chi-squared test with a significance threshold of *P* < .05. This process yielded 5083 (27%) distinct GCFs out of the 18 596 derived from BGC Atlas, and we report these GCFs in Tables 2 and 3, available as supplementary data at *Bioinformatics* online.

**Figure 2:**
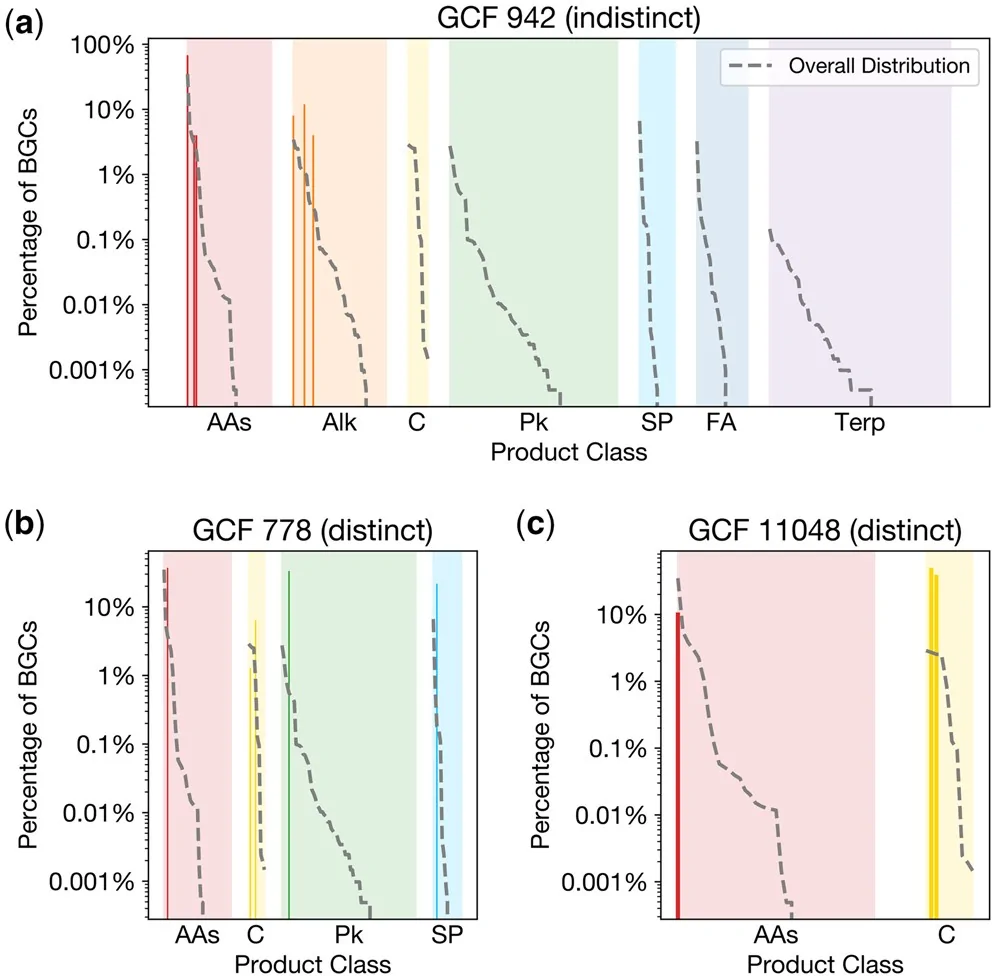
Example PCPs for different GCFs constructed using *BGCat*. (a) A GCF with an indistinct distribution of product types: amino acids and alkaloids are represented similarly to the overall dataset. (b) An example of a GCF with a distinct distribution of product classes featuring a multitude of product types. (c) A GCF with a distinct distribution specialized in specific product classes. In both distinct cases, the proportion of BGCs responsible for a given product type is well above the average for the dataset (dashed line). Classes are grouped into pathways: Amino acids (“AAs”), Alkaloids (“Alk”), Carbohydrates (“C”), Polyketides (“Pk”), Shikimates and Phenylpropanoids (“SP”), Fatty acids (“FA”), and Terpenoids (“Terp”).

Select examples of such PCPs are shown in Fig. 2. GCF 942 (Fig. 2a) is representative of an indistinct GCF, whose product type distribution is not significantly different from the background. The two most common pathways, Amino acids & Peptides and Alkaloids are represented in this family in a similar proportion to the overall dataset. In contrast, GCF 778 (Fig. 2b) has a distinct distribution with a rich variety of product types, with classes from the Carbohydrates, Polyketides, and Shikimates & Phenylpropanoids pathways notably overrepresented. GCF 11 048 (Fig. 2c) is also an example of a distinct GCF, albeit a more specialized one for production of carbohydrates. It should be noted that while most GCFs are associated with only a handful of classes, the entire distribution of product types should be considered as it is both positive and negative predictions that together provide a statistical signal. Since product classification is applied downstream from GCF determination, it adds an additional dimension to the analysis of GCFs by highlighting notable families according to their ultimate biological function that would otherwise be opaque to the clustering method.

### 3.6 *BGCat* predicts detailed product labels for microbial BGCs in antiSMASH DB

To showcase a practical application of our method, we apply it to antiSMASH DB (Blin *et al.* 2023b), a public repository of over 260k BGCs from over 36k bacterial genomes precomputed using antiSMASH. Although antiSMASH is able to elucidate the approximate scaffold of some products, in most cases it is unable to predict the full structure or detailed classification of the product. One way to gather deeper context about the product is to leverage the KnownClusterBlast (KCB) module of antiSMASH that identifies similar clusters in MIBiG and thus can provide structures of related natural products. However, in some cases, the MIBiG BGCs may not include the product structure, or a KCB match may not be available for a particular antiSMASH BGC.

We can therefore separate antiSMASH DB into three subsets. Subset 1 consists of 114k BGCs with both a KCB match and one or more product structures available in the corresponding MIBiG BGC. Subset 2 contains 27k BGCs that have a KCB match but no machine readable structure provided in MIBiG. Subset 3 includes 121k BGCs that feature no KCB matches at all. The first two subsets offer an opportunity to evaluate *BGCat*, since the MIBiG BGCs identified by KCB will contain a coarse classification of the product at minimum; subset 1 additionally provides product structures, which can be used to compare NPClassifier’s performance to the same standard. The final subset offers opportunity for new discovery using our prediction approach. An overview of the three subsets can be found in Fig. 1, available as supplementary data at *Bioinformatics* online.

To evaluate our model, we implement a mapping from NPClassifier terms to MIBiG coarse human-annotated labels and consider the percentage of BGCs for which at least one of *BGCat* predictions is in agreement with the MIBiG classification. This measure effectively represents our model’s ability to recall information from MIBiG BGC labels. These labels, being annotated by human experts, can be considered highly accurate; however, due to individual preferences or unknown BGC products these labels may not necessarily be exhaustive. Most categories map trivially; however, Shikimates and Phenylpropanoids had no direct equivalent and had to be mapped to the MIBiG “Other” category. Fatty acids were mapped to Polyketides in MIBiG due to the close structural and evolutionary similarities between their corresponding synthases (Kohli *et al.* 2016). RiPPs and NRPs are difficult to distinguish based on the peptide structure alone, so they were grouped into one surrogate MIBiG label “RiPP/NRP.” The objective of the model is therefore to predict a detailed product class that maps to the same label found in a MIBiG KCB match.

On subset 1, the top prediction of the model is able to recover a correct MIBiG label for 76% of the BGCs; the percentage increases to 84% when top two predictions are considered. NPClassifier on average also predicts two labels per BGC, and its rate of agreement with MIBiG is similarly at 85%. Because our dataset is based on the product classification from NPClassifier, this result represents the upper bound on the *BGCat*’s accuracy and its ability to nearly match it is encouraging. On subset 2, the machine readable structure of the MIBiG product is not available, which precludes the use of NPClassifier. However, our model is still able to recover at least one MIBiG label for 54% of BGCs with its top prediction, and for 67% of BGCs with its top two predictions. The lower performance is expected in this case because the lack of structure in a MIBiG BGC likely means the structure is not part of the MIBiG database, which would prevent our model from observing it in the training set.

Finally, we consider subset 3, for which no KCB data is available. In this case, *BGCat* can provide unique insights about the products of these BGCs. We offer our top 3 predictions for all of the 121k BGCs as the Table 4, available as supplementary data at *Bioinformatics* online. To evaluate utility of these new results relative to the available GCF groupings, we calculated the Normalized Mutual Information (NMI) between the GCF assignments and the labels. NMI is an established metric for evaluating the agreement between two clusterings based on information theory. For pathway-, superclass-, and class-level labels, the NMI was found to be 0.16, 0.27, and 0.38, respectively. These low values indicate that product labels and GCF membership share relatively little mutual information and therefore capture largely orthogonal aspects of BGC biology: GCFs reflect sequence similarity, whereas *BGCat*’s predictions reflect chemical outcomes. The increasing NMI from pathway to class level further suggests that finer-grained product labels align more closely, but still only modestly, with genomic similarity, whereas coarse labels obscure these relationships. Thus, *BGCat* provides substantial, nonredundant information beyond what GCF groupings alone can offer.

**Figure 3:**
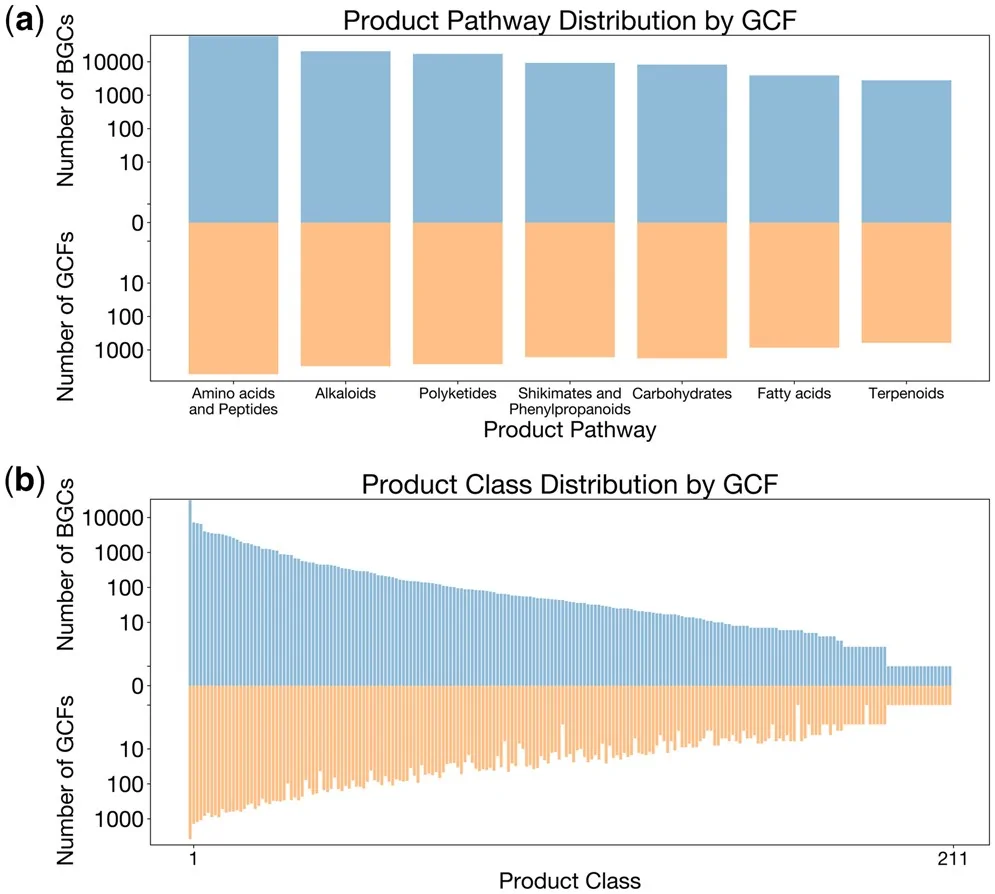
Distributions of BGCs and GCFs in the antiSMASH DB subset 3, stratified by: (a) the seven pathway labels, and (b) product class labels. The *x*-axis represents different product labels predicted by the model. The *y*-axis represents the number of BGCs or GCFs which contain a given product class. The BGCs are shown as  bars in the upper half of each plot, while the GCFs are shown as  bars in the bottom half. These distributions highlight the broad biochemical coverage of subset 3 and reveal substantial fine-grained diversity, underscoring the value of *BGCat’*s product-based predictions for characterizing BGCs beyond sequence-based groupings.

Figure 3 illustrates the breadth and diversity of product types predicted across antiSMASH DB subset 3. At the pathway level (Fig. 3a), all seven natural product pathways are well represented, with Amino acids & Peptides and Polyketides dominating both BGC and GCF counts. This broad coverage suggests that *BGCat* predictions span the full metabolic landscape of microbial natural products. In contrast, the class-level distributions (Fig. 3b) exhibit a characteristic long-tail pattern: a handful of classes contain thousands of BGCs, whereas many others are sparsely populated. This imbalance underscores both the chemical richness of BGC-encoded metabolites and the difficulty of fine-grained classification. Notably, GCF counts broadly track BGC frequencies, indicating that highly populated classes correspond to genuinely diverse biosynthetic families rather than duplicated clusters. In other words, classes with many BGCs also contain many distinct genomic architectures, showing that their abundance reflects true biosynthetic diversity rather than repeated instances of the same cluster. It would be interesting to see if this pattern holds for other methods; however, there is currently no other approach that uses the NPClassifier nomenclature to compare against. Together, these figures validate the utility of fine-grained structural classification and highlight the data challenges inherent to learning at this level of resolution.

## 4 Conclusions

*BGCat* is a deep neural network for fine-grained BGC product classification. It builds upon the NPClassifier (Kim *et al.* 2021) nomenclature for natural products, making it particularly suitable for describing metabolites arising from BGCs. By leveraging a large pre-trained protein language model, our method takes advantage of the vast repository of available protein sequence information to create biologically meaningful representations of biosynthetic genes. In addition, our novel data augmentation strategy allows the neural network to learn from a greater variety of BGCs than would otherwise be possible with presently existing datasets. We show that our method is effective both for traditional coarse-grained as well as fine-grained BGC product classification. The fine-grained approach is especially interesting in the context of BGC products as there is currently no method capable of predicting the complete structure of such molecules in the general case; thus, detailed product classification offers the greatest insight into the function of BGCs currently available. Furthermore, we anticipate that *BGCat* can improve paired omics analyses by improved linking and ranking of predicted BGCs in genomes to mass spectra with predicted compound classes (van der Hooft *et al.* 2020).

In contrast to prior algorithmic methods, our machine learning-based approach aims to be generalizable to different types of clusters and products. We have shown that the model is effective for predicting product classes for new BGCs introduced over time and maintains a degree of accuracy on novel types of BGCs when grouped by GCFs. As new information about BGCs becomes available, our model can be trained on new data to further enhance its accuracy and coverage of product types. It should be noted, however, that our model may need further adaptations to apply beyond microbial BGCs, as BGCs found in other organisms often require special considerations (Kautsar *et al.* 2017, Kwon and Hovde 2024, Del Pup *et al.* 2026).

To highlight a practical application of our method, we have used it to provide product labels for a diverse set of microbial BGCs deposited in antiSMASH DB. We have shown that our model provides product class labels in agreement with MIBiG labels for at least 54% of BGCs with KCB matches. However, of particular interest was the subset of 121k BGCs with no KCB matches: in this case, our model provided the only means of predicting the BGC product type.

While our tool was effective in different BGC product classification scenarios, it has a few limitations that could be addressed in future work. Firstly, the method was trained on a restrictive dataset derived from the core MIBiG dataset of 2046 BGCs, which provides limited sequence and product diversity for the model to learn from. To alleviate this issue, we introduced a data augmentation scheme that related additional BGC Atlas sequences to MIBiG clusters. This approach relies on the assumption that all BGCs within a given GCF produce substantially similar products, which may be more valid for certain types of BGCs than others, e.g. those containing more conserved backbones versus tailoring enzymes. Although these augmented BGC-product class relationships are less reliable than the human-curated MIBiG annotations, we observed that their inclusion in the dataset has been beneficial. It stands to reason that expanding the dataset—whether with additional high-quality curated labels or augmented “noisy” labels—would further enhance the model. Secondly, our method relies on NPClassifier predictions for labeling products in the dataset, which uses a deep neural network that may not always yield the correct results. Unfortunately, there is currently no manually curated dataset that provides detailed classification of BGC products; however, we anticipate that this situation will improve over time. Finally, the ESM Cambrian model was originally trained on protein sequences and as such may not be ideal for embedding BGC genes. Future work may consider the use of fine tuning or end-to-end training of the language model on genomic datasets to improve its match to tasks involving biosynthetic genes.

However, despite these limitations, our technique remains effective for detailed BGC product classification and is the first one to emphasize the importance of using a nomenclature designed for natural products. By using a fine-grained system for describing product types, *BGCat* offers an unmatched insight into the products of individual BGCs and GCFs in aggregate. We expect that this additional context will lead to advances in product structure prediction and, more broadly, an improved understanding of BGC functions.

## Supplementary Material

btag559_Supplementary_Data

## Data Availability

The data used in this work is based on publicly available datasets. The MIBiG datasets are available at https://mibig.secondarymetabolites.org. The BGC Atlas dataset can be downloaded from https://bgc-atlas.cs.uni-tuebingen.de. AntiSMASH DB is available at https://antismash-db.secondarymetabolites.org. Our trained model and inference code are available at https://github.com/HassounLab/BGCat.
