# Supplementary material for: Fine-grained structural classification of biosynthetic gene cluster-encoded products
Source: Bioinformatics. 2026 Aug 17;42(8):btag559. doi: 10.1093/bioinformatics/btag559 (PMC13600415; doi:10.1093/bioinformatics/btag559)
Supplement: Supplementary file 1 [file btag559_supplementary_data.zip › BGCat-supplementary.pdf]

Fine-Grained Structural Classification of Biosynthetic Gene Cluster-Encoded Products

Vladimir Porokhin[1], Emily Mevers[2], Justin J. J. van der Hooft[3,4], Soha Hassoun[1,5,*]

[1] Department of Computer Science, Tufts University, Medford, MA 02155, United States
[2] Department of Chemistry, Virginia Tech, Blacksburg, VA 24061, United States
[3] Bioinformatics Group, Wageningen University & Research, the Netherlands
[4] Department of Biochemistry, University of Johannesburg, South Africa
[5] Department of Chemical and Biological Engineering, Tufts University, Medford, MA 02155, United States
[*] Corresponding author. Email: soha.hassoun@tufts.edu

Supplementary Materials

This section lists all supplementary files referred to in the main text and included in the zip archive.

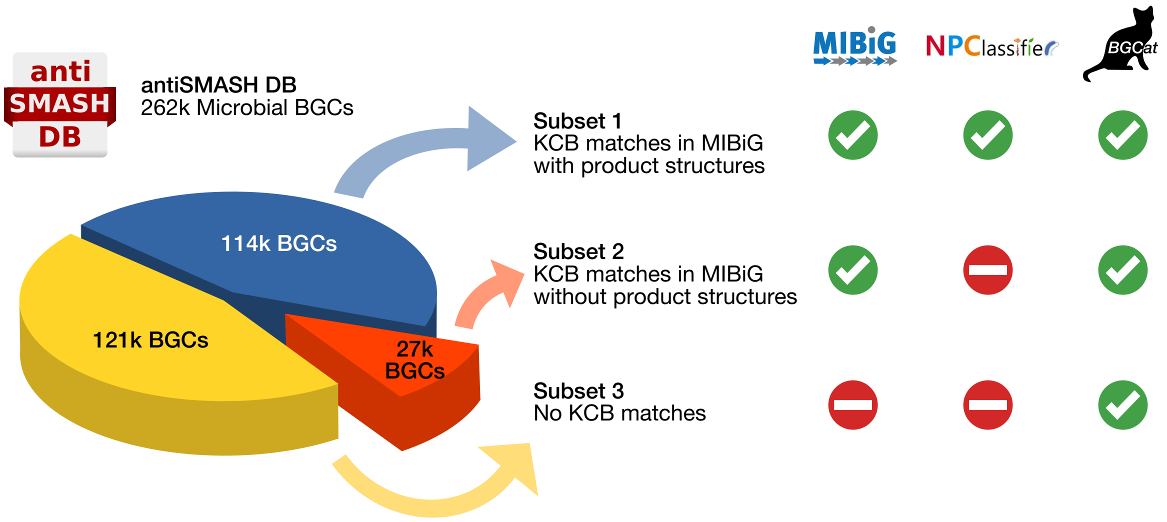

Supplementary Figure 1: An overview of the three antiSMASH DB subsets. BGCs from subset 1 feature KCB matches to MIBiG BGCs with product structures, allowing the use of MIBiG labels and NPClassifier predictions for BGC characterization. Subset 2 also has matches to MIBiG BGCs, albeit without machine-readable product structures, precluding the use of NPClassifier. Finally, subset 3 features no KCB matches at all, which eliminates the availability of MIBiG labels as well. However, *BGCat* remain available for all three subsets.

Supplementary Table 2. List of BGC Atlas BGCs and their corresponding GCFs. Predicted product class labels are also included for each BGC.

Supplementary Table 3. List of GCFs generated based on BGC Atlas, indicating whether or not a given family has a distinct PCP. In addition, labels for the most overrepresented product classes are included for each GCF.

Supplementary Table 4. List of antiSMASH DB BGCs from subset 3 that lack KCB hits in MIBiG. For each BGC, we provide the top 3 *BGCat* product classification predictions.

Supplementary Table 1: NP label prediction performance without (a) and with (b) class weighting for the realistic data split with data augmentation.

Label type	Micro averaging				Macro averaging			
	AP	AUROC	Recall	Precision	AP	AUROC	Recall	Precision
a) Realistic split without class weighting								
All labels	0.315	0.835	0.271	0.531	0.112	0.646	0.049	0.455
Pathway	0.527	0.790	0.505	0.530	0.403	0.734	0.367	0.443
Superclass	0.304	0.807	0.242	0.562	0.129	0.691	0.067	0.386
Class	0.151	0.772	0.127	0.448	0.094	0.628	0.032	0.452
b) Realistic split with class weighting								
All labels	0.285	0.848	0.309	0.413	0.103	0.606	0.093	0.202
Pathway	0.535	0.789	0.499	0.518	0.428	0.743	0.397	0.436
Superclass	0.258	0.811	0.256	0.410	0.124	0.667	0.095	0.250
Class	0.150	0.768	0.184	0.348	0.086	0.588	0.062	0.247
